# A cross-sectional serosurvey in a sheep population in central Italy following a bluetongue epidemic

**DOI:** 10.1371/journal.pone.0208074

**Published:** 2019-01-09

**Authors:** Andrea Carvelli, Marcello Sala, Gian Luca Autorino, Maria Teresa Scicluna, Francesca Iacoponi, Pasquale Rombolà, Paola Scaramozzino

**Affiliations:** 1 Epidemiology Unit, Istituto Zooprofilattico Sperimentale del Lazio e della Toscana “M. Aleandri”, Roma, Italy; 2 Virology Unit, Istituto Zooprofilattico Sperimentale del Lazio e della Toscana “M. Aleandri”, Roma, Italy; Faculty of Science, Ain Shams University (ASU), EGYPT

## Abstract

Bluetongue (BT) is a viral disease that affects ruminants and is transmitted by midges of the genus *Culicoides* spp. The seroprevalence, the clinical form and the occurrence rates significantly differ in relation to several factors such as bluetongue virus (BTV) serotype, host species, breed susceptibility, specific previous exposure, vector ecology, husbandry and health status. Following the 2001–2006 BTV2 and BTV16 epidemics in central Italy, a new epidemic caused by BTV1 occurred in 2013–2015 causing 398 outbreaks in a susceptible population of about 1 million ruminants. The present study assessed the BTV1 seroprevalence in the sheep population of central Italy by conducting two cross-sectional surveys, in the proximity of and within BT outbreak farms. A total of 2,984 sheep from 437 farms were sampled. The animal-level prevalence was 19% (95% CI: 17–21%), the between-herd prevalence was 46% (95% CI: 41–51%) and the within-herd prevalence was 21% (95% CI: 16–26%). Risk factors were investigated by logistic regression models. Living on a farm where an outbreak occurred and the number of outbreaks in proximity of the farm were identified as risk factors, while herd size was identified as a protective factor. This study represents the first BT survey in southern Europe and reports valuable findings on BTV epidemiology. Despite intensive virus circulation, the estimated seroprevalences were low. The assessment of the population immunity level is crucial for defining an efficient vaccination strategy and for predicting the impact of future virus circulation. In view of the low seroprevalence detected albeit an extensive BTV1 circulation, the population immunity was likely to be inadequate in preventing new BTV1 epidemics. Moreover, considering the recurrent introduction of new serotypes from North Africa and the Balkans, the control of multi-serotype BTV infections will continue to present a challenge in the near future.

## Introduction

Bluetongue (BT) is a viral disease that affects domestic and wild ruminants, transmitted by midges of the genus *Culicoides* spp. and is a notifiable disease according to the European Union legislation and OIE regulations [1]. In recent years, novel putative serotypes were detected further to the 24 traditional serotypes, adding up to 30 different serotypes [1]. The infection with a BT virus (BTV) confers immunity to the same serotype with no or weak protection against the other serotypes [2]. Bluetongue is an important disease that has severe direct and indirect economic consequences. The direct costs depend on the production losses caused by the clinical disease, while expenditure due to vaccination and lost revenue because of the animal trade restriction are the indirect costs [3]. The impact of clinical disease in terms of morbidity, mortality and case fatality rates significantly differs in relation to the several factors involved in the disease epidemiology such as BTV serotype, ruminant species infected, breed susceptibility, previous population exposure, vector ecology, husbandry and individual health status [1]. Nevertheless, the main economic impact of BT is due to the national and international trade restrictions of ruminants from the infected zones to the disease-free areas (European Commission Regulation No 1266/2007 of 26 October 2007) [3].

Since 2000, Italy experienced repeated BT incursions. Seven serotypes (BTV1, BTV2, BTV3, BTV4, BTV8, BTV9, BTV16) circulated within the country with different epidemiological patterns.

In the Lazio region (central Italy), the spread of BTV2 and BTV16 occurred between 2001 and 2006. After 7 years of epidemiological silence, a BTV1 of North Africa origin [4] caused a new epidemic in August 2013 continuing up to 2015 [5]. In Italy, an intensive surveillance system is in place since 2001, with the aim of detecting the possible BTV incursions and monitoring the spatial and temporal spread of the existing BTV serotypes and vectors [6]. The surveillance system is based on three components: passive clinical surveillance (notification of clinical suspect cases by farmers and veterinarians), serological surveillance (monthly testing of sentinel cattle and small ruminants) and entomological surveillance (a weekly *Culicoides* trap collection per province). In the Lazio region, 655 sentinel animals on 76 farms are serologically tested for BTV on a monthly basis. A positive result in the screening test must be confirmed by the virus neutralisation test (VNT), which also allows the identification of the BTV serotype involved. In addition, the clinical suspects are investigated using a serotype-specific Real Time Reverse Transcriptase PCR (RT PCR). During 2013/2014 surveillance activities, 398 outbreaks were detected in the Lazio region (clinical, virological and serological positive cases), distributed in 171 municipalities (Table 1). All the Lazio municipalities (378) were put under sanitary restriction measures through the set-up of the Restriction Zone, as prescribed by the EU Directive 2000/75/EC. According to the National Animal Disease Notification System (Siman: Sistema Informativo Nazionale Malattie Animali) [7], the measures of disease occurrence in sheep during this period were: 5.4% morbidity rate, 3.6% mortality rate and 65.9% case fatality rate (Table 1).

**Table 1:** Bluetongue susceptible animal population, number and type of outbreak and measures of disease occurrence in Lazio region in 2013/2014.

Province†	Ruminants‡			No of outbreaks			Occurrence rates (sheep, clinical outbreak)		
	Herds	bovine, buffalo	sheep, goat	Clinical outbreak^§^	Seroconversion*	Virus detection**	Morbidity (%)	Mortality (%)	Case fatality (%)
FR	9,510	53,162	75,748	78	17	2	8.5	5.6	65.8
LT	2,630	89,979	48,109	5	19		4.6	2.9	62.2
RI	4,012	29,843	78,990	66	9		5.1	3.2	62.9
RM	5,257	64,857	256,834	75	41	3	4.9	3.6	72.9
VT	2,567	35,605	323,757	65	17	1	4.7	2.9	60.7
Lazio region	23,976	273,446	783,438	289	103	6	5.4	3.6	65.9

†: FR = Frosinone; LT = Latina; RI = Rieti; RM = Roma; VT = Viterbo.

‡: bovine, buffalo, sheep, goat.

§: a farm where an official veterinarian notified an outbreak in Siman on the basis of the following definition: an animal belonging to a susceptible species presenting a clinical sign consistent with the presence of bluetongue, confirmed by at least one molecular assay (RT PCR).

*: a sentinel animal with a cELISA negative test followed by a cELISA positive test, confirmed by virus neutralisation test.

** Virus detection: detection of virus or viral genetic material in blood or in a tissue sample by RT PCR.

The *Culicoides obsoletus* group and *C*. *imicola* were the vectors mainly involved during both the 2001–2006 and the 2013/2014 epidemics [8]. In the recent epidemic, the majority of notified outbreaks occurred inland, mainly in woodland, where the *C*. *obsoletus* group is considered as the main vector population and *C*. *imicola* is largely absent [9, 10].

When infecting the ruminant host, BTV produces a viraemia that varies in the different ruminant species in terms of virus titres and duration. The viraemic period lasts up to 10 days in sheep [11] and it can last up to 60 days in cattle. The long duration of viraemia in cattle identifies this species as the BTV amplifying host and reservoir [12]. Infected ruminants produce both cell-mediated and humoral immune responses, producing high BTV antibody titres. Serotype-specific neutralising antibodies directed against viral protein (VP) 2 are detected by the VNT, while antibodies directed against VP7 are detected by serogroup-reactive assays such as competitive enzyme-linked immunosorbent assay (cELISA) [13]. Viral protein 2 induces serotype-specific neutralising antibodies that confer a very limited or null protection against the other serotypes [2]. Antibodies against BTV can persist from 2 weeks to up to 6 years after infection [14].

A few cross-sectional surveys have been performed to determine the population immunity level against BTV after field virus circulation [15]. Most of these studies were conducted in tropical countries where the warm climate is optimal for the vector activity and the environmental conditions are suitable for BT endemicity [16, 17, 18, 19]. In Europe, surveys were especially carried out to assess the seroprevalence and to evaluate the efficacy of vaccination campaigns for BTV8. This serotype denoted a different epidemiological behaviour with respect to the other serotypes. It was capable to be vertical transmitted through a trans-placental infection, it overwintered at high latitudes (where BT was never recorded before) and it caused clinical cases in cattle. Due to the attention dedicated to this serotype, BTV8 and studies in cattle are over-represented in surveys carried out in Europe [20].

A systematic literature review on the prevalence of BT in Europe from 2000 to 2010 was carried out by the European Food Safety Authority (EFSA), consulting 80 relevant publications, originated from nine European countries (Belgium, Bulgaria, Denmark, France, Germany, Italy, Netherlands, Sweden and Switzerland), mostly based on antibodies detection [20]. The review reported that BT median prevalence in small ruminants ranged from 20% to 62% (minimum: 0.6%, maximum: 88%) at animal-level and from 50% to 70% (minimum: 7%, maximum: 95%) at herd-level.

The use of vaccination is one of the strategies for the control and prevention of BT [21]. Both live and inactivated vaccines are available generating an effective immune response to structural and non-structural viral proteins. To date, no commercial vaccine producing antibodies differentiating infected from vaccinated animals (DIVA) is available [22]. Since 2008, the use of modified live vaccines ceased in Europe due to problems such as reassortment between vaccine and field viruses, adverse effects during pregnancy and diffusion of vaccine strains by midges [23]. Nevertheless, the cost of inactivated vaccines is high as a result of production costs and due to the repeated boosters required to achieve an adequate immunity level [24]. The cost-effectiveness of a mass vaccination campaign should be carefully evaluated, especially when economic resources are limited. In this view, the level of population immunity should be taken into account when the competent veterinary authorities are in charge of planning vaccination policies. The scenario can be further complicated by possible successive incursions of different BTV serotypes against which there is limited or null cross-immunity deriving from previous serotype-specific vaccination, both at individual and population level [2]. In particular, during 2013/2014, in the Lazio region, a few BTV1 vaccination interventions were performed exclusively on the farmers’ request, especially in cattle for trade purposes, thus leaving the local population naïve.

The purpose of this study was to assess the BTV prevalence in a sheep population in central Italy after a BTV1 epidemic. In view of the limited available doses of vaccine and the high number of susceptible animals eligible for vaccination, the competent authorities requested a prioritization of vaccine interventions. A multi-step approach was designed to provide useful evidence to support the decisions on vaccination strategies, by assessing BTV seroprevalence in sheep in a worst-case scenario. Two areas most likely exposed to BTV1 circulation were targeted, assuming that exposure of sheep to BTV infection in the rest of the region was lower. An Around-Outbreak (AO) survey was performed in the municipalities where BTV circulated, excluding outbreaks sites. An In-Outbreak (IO) survey was conducted within farms where a clinical outbreak was notified by the competent veterinary authorities.

Furthermore, variables such as use of land (Corine Land Cover, CLC) [25], altitude, herd size, ruminants species density and number of outbreaks in the proximity of the herds were investigated to assess if these factors increased the likelihood of an animal being BTV seropositive.

## Materials and methods

### BTV circulation in the study area

Data on BTV susceptible population (animals and herds) in the Lazio region were obtained from the National Animal Registry Database (Banca Dati Nazionale: BDN) [26]. Bluetongue outbreaks were notified in Siman by competent veterinary authorities [7]. Occurrence rates were calculated from the data registered in Siman, where official veterinarians of the Local Health Units recorded the number of BT cases and the number of dead animals as requested by the National Regulation [27] (Table 1). As a Regional Act ensured recompensation of dead animals after a BTV RT PCR positive test, under and over-reporting of BT cases was considered unlikely to have occurred.

The amount of movements and trade of live animals that occurred during the study were considered negligible.

The kernel smoothing function implemented in ArcGis 10.3 (ESRI, Redlands, CA, USA) was used to produce the raster maps of cattle and small ruminants’ density (1 km^2^ cell size and 5 km as the bandwidth) (S1 File).

Corine Land Cover [25] was used to classify the use of the land in a 1 km buffer radius around the farms where animals were sampled. The area of each polygon was calculated as a proportion of the total area inside the buffer. The land cover assigned to each animal was the highest value (%) of a category inside the buffer.

### Serosurvey design

**Fig 1:**
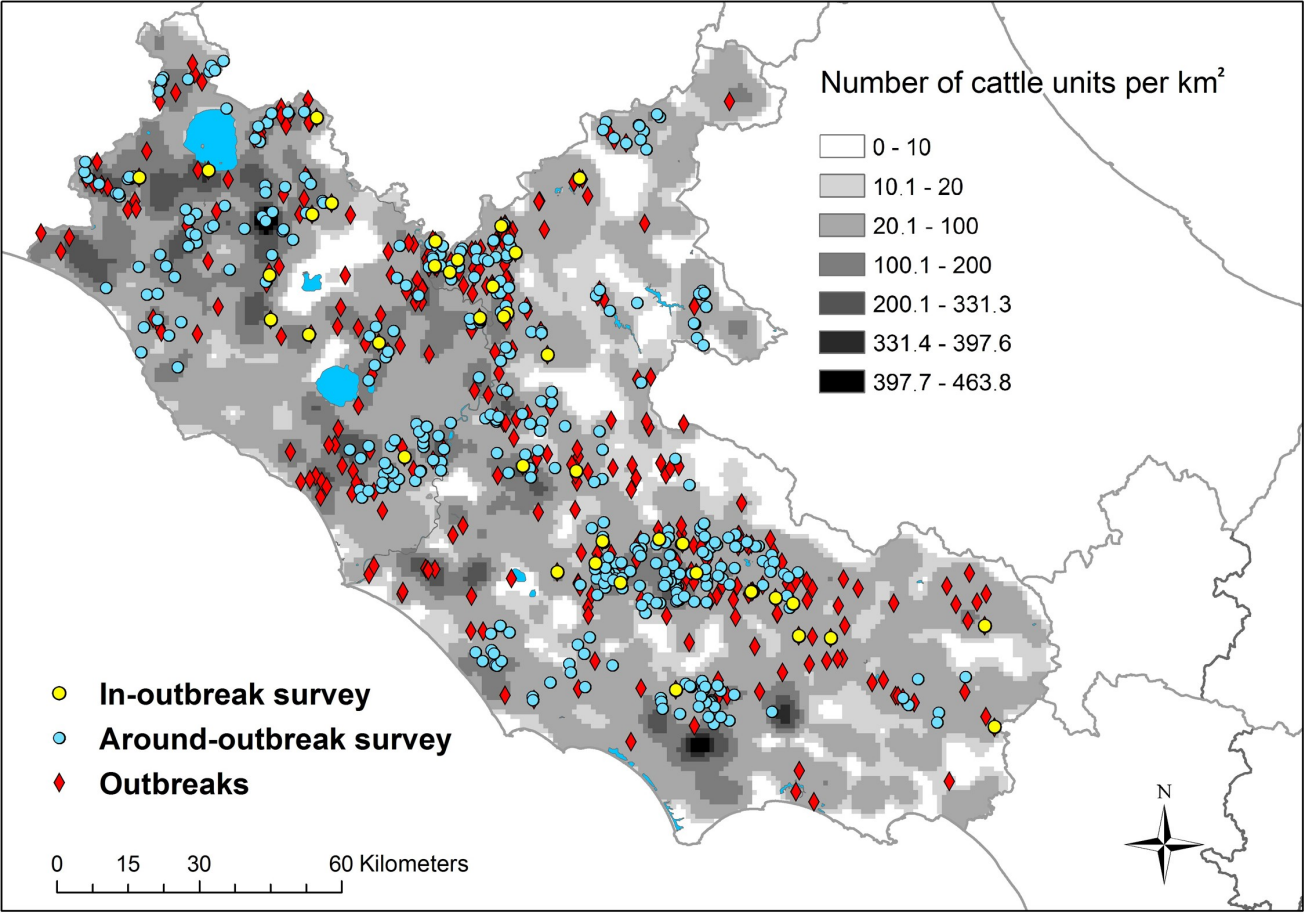
Location of bluetongue outbreaks, IO herds, AO herds and number of cattle units per km^2^ in the Lazio region in 2013/2014.

**Fig 2:**
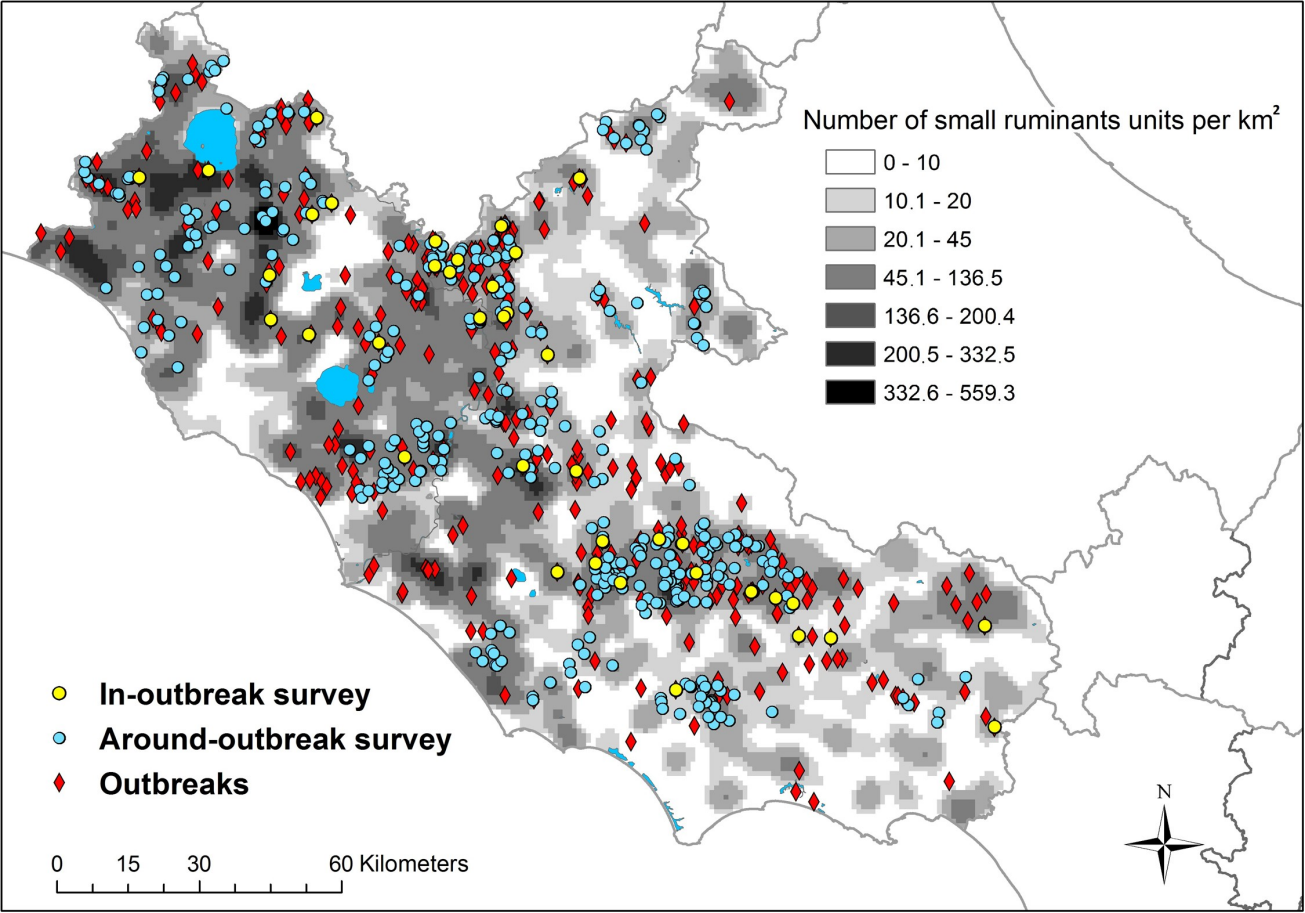
Location of bluetongue outbreaks, IO herds, AO herds and number of small ruminant units per km^2^ in the Lazio region in 2013/2014.

The seroprevalence in the sheep population was estimated by conducting two cross-sectional surveys: AO and IO. Around-Outbreak was performed in an area where BTV circulated, excluding the outbreak farms. In-Outbreak was carried out within farms where an official clinical outbreak was notified by the competent authority (Figs 1 and 2).

In the AO survey the observed prevalences were considered as: animal-level prevalence, defined as the proportion of indirect cELISA positive animals out of the total number of animals tested in the study area and the between-herd prevalence intended as the proportion of cELISA positive herds out of the total number of tested herds in the area. A farm was classified positive if at least one animal was found cELISA positive.

In the IO survey, the prevalence considered was the within-herd prevalence, defined as the proportion of indirect cELISA positive animals from the total number of tested animals in the herd.

Blood samples were collected by official veterinarians of Local Health Units. The sampling procedures and the laboratory tests were performed from December 2014 to February 2015.

The sampling frame was established using sheep farm ID and herd size obtained from the BDN [26]. Herds and animals within the herds were selected by a simple random sampling method. Animals younger than 12 months and older than 5 years were excluded from the sampling frame to respectively avoid detection of antibodies due to maternal immunity [15] and those originating from the 2001–2006 BTV epidemics.

### Around-outbreak survey

The target area included the municipalities where at least two outbreaks (clinical outbreak, seroconversion or positive RT PCR) were notified in 2014. Considering an unknown (50%) *a priori* prevalence, assuming a 3% standard error and a 99% confidence level, the requested sample size was 1843 sheep. The sample size was stratified proportionally to the animal population size/municipality. Four animals from each farm were tested. Farms where BT vaccination was performed and where an outbreak of BT was notified in 2013 and 2014 were excluded.

### In-outbreak survey

A two-stage cluster sampling was performed to assess the within-herd seroprevalence, with herds as primary units and animals as secondary units. The target population was represented by sheep farms where a BT clinical outbreak was notified in 2014. Farms where BT vaccination was performed were excluded. The following assumptions were used: a confidence level of 95%, a standard error of 10%, an unknown (50%) *a priori* prevalence and an intra-cluster correlation coefficient of 0.4. The number of animals to be collected in each cluster was set to 30 animals, resulting in a sample size of 1,210 sheep distributed in 40 clusters. As outbreaks were not homogenously distributed in the Lazio region, to provide a representative sample, herds were stratified proportionally to the number of clinical outbreaks per province.

### Diagnostic methods

Sera were tested using a cELISA kit provided by the Bluetongue National Reference Laboratory [28], capable of detecting antibodies directed against the VP7 of all BTV serotypes. The presence of other serotypes was excluded by performing serotype-specific tests (VNT and RT PCR) in the framework of the routine surveillance activities. The cELISA test had a sensitivity (Se) of 100% (95% CI: 99.7% - 100%) and a specificity (Sp) of 98.4% (95% CI: 97.8% - 98.9%). The cELISA test was carried out in accordance to the instruction of the manufacturer and according to the OIE Manual of Diagnostic Tests. The diagnostic assay classifies samples as positive, equivocal and negatives, however, in a conservative manner, samples with equivocal results (N = 1) were reclassified as negative.

### Statistical analysis

The true prevalence (TP) was calculated from the apparent prevalence (AP) using the Rogan and Gladen equation. The formula for the calculation is:

TP = (AP + Sp– 1)/(Se + Sp– 1) [29].

The TP and Blaker’s 95% confidence intervals (CI) [30] were calculated using the Epitools epidemiological calculator [31]. In the IO survey, the overall animal-level prevalence was also calculated using the herd size as the weighting factor [32]. As the herds were selected by simple random sampling, with a fixed number of animals sampled in each herd, not proportional to the herd size, adjustment of prevalence estimates (P_adj_) and 95% CI were performed using weights to account for the cluster sampling design employed. The formula for the calculation is the following: P_adj_ = ∑ (M_h_/∑M_h_) P_h_, where P_h_ is the proportion of positives individuals in each selected herd (No positive/No tested), M_h_ is the number of animal present in each selected herd and ∑M_h_ is the total number of animal present in all the tested herds.

The following individual exposure variables were considered for the univariable and multivariable analysis: altitude (meters), land use (agricultural, woodland and semi-natural and urban areas, according to CLC) [25], survey design (AO, IO), herd size, cattle population size inside a 1-km-buffer around the sampling farm (hereafter called cattle density), small ruminant population size inside a 1-km-buffer around the sampling farm (hereafter called small ruminant density) and number of BT notified clinical outbreaks inside a 1-km-buffer around the sampling farm. The radius size was chosen in view of the most likely *Culicoides* flying range<1km [33]. Herd size and number of animals were divided into classes following the quartile classification method: herd size (≤60, 61–160, 161–386, ≥387), cattle density (≤4, 5–33, 34–117, ≥118), small ruminant density (≤214, 215–470, 471–919, ≥920). To evaluate linearity, categories were created and univariable analysis was performed. The log odds ratios (ORs) were plotted against the midpoint of the category [34].

The effect of the exposure variables on individual seropositivity was analysed using univariable logistic regression models. Significant variables in the univariable analysis were screened for pair-wise collinearity or associations using Pearson’s correlation coefficient or chi-square test for continuous or categorized variables, respectively.

After excluding associated variables, the risk factors with a bivariate p value ≤0.25 were included in a multivariable stepwise logistic mixed model with herds as random effect [35]. The final model was evaluated by the likelihood ratio chi square test (LR).

A p value <0.05 was considered statistically significant. Data are presented as frequencies, percentages (%), medians and interquartile ranges (75°-25° percentile) depending on the variable distribution. All statistical analyses were performed by Stata/SE version 12 for Windows (StataCorp LP, TX, USA).

### Ethical statement

Serum samples were collected by Local Health Units official veterinarians in accordance with the European Legislation on Animal Welfare. Approval of an ethics committee was not required for sampling because a single blood collection sample is considered a routine procedure in domestic animals. Anaesthesia, euthanasia or any kind of animal sacrifice were not part of the study.

## Results

### BTV circulation in the study area

According to the BDN [26], in 2014 the BTV susceptible population in the Lazio region was 1,056,884 ruminants (26% cattle and buffalo—*Bubalus bubalis*; 74% sheep and goats) housed in 23,976 farms in five provinces (FR: Frosinone; LT: Latina; RI: Rieti; RM: Roma; VT: Viterbo). The spatial distribution of ruminant species in Lazio is shown in Figs 1 and 2.

Before the present survey, 398 outbreaks (clinical cases, seroconversion and virus detection) were notified in 2013/2014. The clinical outbreaks in sheep represented 72.6% of these outbreaks (Siman) and were confirmed as positive using a RT PCR. As the Lazio region covers a surface of 17,203 km2, 2.3 outbreaks/100 km2 were notified.

### Around-outbreak survey

The number of municipalities with at least two BT outbreaks in 2014 was 77 (out of 378). In these municipalities, 285 outbreaks were notified in Siman and the number of sheep farms was 2,777 with a population of 275,743 animals [26]. A total of 1,779 sheep from 397 herds were sampled and tested for BT. The animal-level true prevalence was 19% (Blaker’s 95% CI: 17–21%), with a range of 5% to 32% in the different provinces. The between-herd prevalence was 46% (95% CI: 41–51%), varying from 21% to 64% in the different provinces (Table 2).

**Table 2:** The seroprevalence of bluetongue in the sheep population.

Province†	No outbreak/100 Km^2^	Survey AO				Survey IO		
		Herds		Animals		Herds	Animals	
		Tested	% positive (CI 95%)	Tested	% positive (CI 95%)	Tested	Tested	% positive (CI 95%)
FR	3.0	59	62 (49–74)	233	32 (26–39)	11	331	31 (26–36)
LT	1.1	52	49 (35–63)	310	18 (14–23)	1	30	29 (14–48)
RI	2.7	76	64 (52–74)	308	29 (24–35)	10	299	42 (36–47)
RM	2.2	129	43 (34–52)	603	15 (12–19)	9	275	22 (17–28)
VT	2.3	81	21 (12–31)	325	5 (3–9)	9	270	7 (4–11)
Lazio region	2.3	397	46 (41–51)	1,779	19 (17–21)	40	1,205	26 (23–29)

†: FR = Frosinone; LT = Latina; RI = Rieti; RM = Roma; VT = Viterbo.

### In-outbreak survey

According to the eligibility criteria, the number of sheep farms where at least one clinical outbreak was notified in 2014 was 275. A total of 1,205 sheep from 40 farms were sampled and tested for BT. The within-herd prevalence was 26% (Blaker’s 95% CI: 23–29%), ranging from 7% to 42% in the different provinces (Table 2). The weighted animal-level prevalence was 21% (Blaker’s 95% CI: 16–26%).

### Risk factors analysis

Univariable and multivariable analyses were performed. In the univariable analysis (Table 3), woodland and semi-natural areas were defined as risk factors, while living in urban areas was not significant when compared to the baseline value. We observed a significant difference in the individual likelihood of being positive in IO with respect to AO (OR = 1.50). Our results identified herd size as a protective factor. An increase of animals inside the farm determined a decrease of risk of being positive. The density of small ruminants was a significant protective factor for the class >920 animals (OR = 0.51), while cattle density was a significant risk factor for the classes ≥34 animals (OR = 1.66 in 34–117 class, OR = 1.43 in ≥118 class). The number of outbreaks in a 1-km-buffer was another significant risk factor (OR = 1.21). No significant risk difference in farms altitude (p = 0.27) was found.

**Table 3:** Univariable analysis for the exposure variables relative to the animal seropositivity outcome.

	Negative (N = 2296)	Positive (N = 680)	OR (95% CI)	p value
Altitude (m)	272.93 (195.81)	263.82 (170.13)	0.99 (0.99–1.00)	0.273
Land use (CLC)				
Agricultural	2097	590	-	
Woodland and semi-natural	146	74	1.80 (1.34–2.42)	<0.001
Urban	53	16	1.07 (0.61–1.89)	0.807
Survey design				
AO	1418	353	-	
IO	878	327	1.50 (1.26–1.78)	<0.001
Herd size				
≤60	450	199	-	
61–160	561	187	0.75 (0.60–0.95)	<0.05
161–386	576	127	0.50 (0.39–0.64)	<0.001
≥387	609	118	0.44 (0.34–0.57)	<0.001
Cattle density				
≤4	643	147	-	
5–33	557	146	1.15 (0.89–1.48)	0.294
34–117	536	204	1.66 (1.31–2.12)	<0.001
≥118	560	183	1.43 (1.12–1.83)	<0.01
Small ruminants density				
≤214	551	193	-	
215–470	567	188	0.95 (0.75–1.19)	0.644
471–919	546	187	0.98 (0.77–1.23)	0.850
≥920	632	112	0.51 (0.39–0.66)	<0.001
No of outbreaks	2.36 (2.28)	3.62 (2.80)	1.21 (1.17–1.25)	<0.001

The cattle and small ruminants densities were excluded from the multivariable model since they were significantly associated (p<0.001) with the herd size. The risk factors identified in the multivariable logistic model (LR = 179.11, p<0.001) were: to live on farms where outbreaks were notified (IO survey) and the number of BT outbreaks in a 1-km-buffer. The herd size was confirmed as a protective factor (Table 4).

**Table 4:** Multivariable analysis for the exposure variables relative to the animal seropositivity outcome.

Variable	OR	IC 95%	p value
Survey design			
AO	-		
IO	1.95	1.23–3.09	<0.01
Herd size			
≤60	-		
61–160	0.65	0.41–1.03	0.065
161–386	0.37	0.22–0.62	<0.001
≥387	0.39	0.21–0.61	<0.001
No of outbreaks	1.26	1.18–1.36	<0.001

## Discussion

During the BT epidemic which occurred in 2013/2014 in the Lazio region, the susceptible animal population was completely exposed to BTV1 infection. An efficient vaccination strategy or an evaluation of the possible impact of future virus circulation without vaccination depends on an assessment of the population immunity level. The European Food Safety Authority recently published that the eradication of BT could be managed, only if specific conditions (e.g. animal density, meteorological conditions, animal husbandry) are considered in a case-by-case approach [15].

The 2013/2014 BTV1 epidemic in central Italy occurred after years of absence of BT as previous BTV circulation occurred in the same area from 2001 to 2006 due to BTV2 and BTV16 [5]. Thus, a novel serotype of North African origin was introduced in a naïve population of central Italy [4]. A huge variability in morbidity and mortality rates–i.e. from <5% to 100%–was reported in different sheep populations depending on many factors such as the virus strain, the general health status, age and breed of the host and the vector population species, competence and abundance [15]. In the Lazio region, the 2013/2014 morbidity and mortality rates were 5.4% and 3.6%, respectively. These rates were lower than expected, considering that the animal population was completely naïve, but similar to the rates observed for the same BTV1 strain in Sardinia and in North Africa (Siman, OIE WAHIS). During the BTV2 epidemic in 2001–2003, in the same area, the registered occurrence rates were even lower, 1.87% morbidity and 0.29% mortality [36], suggesting low direct impact losses. For this reason, animal breeders’ associations did not consider BT as an important disease and were therefore reluctant to vaccinate animals.

The present study assessed the seroprevalence of BTV1 after one year of virus circulation in an area where density of domestic ruminants was 61.29 animal/km^2^ and where 2.3 BT outbreaks/100 km2 were notified. The animal-level and the within-herd prevalences were low, 19% and 21%, respectively. The between-herd prevalence (46%) estimated in the AO survey was biased because only four animals per farm were tested and, therefore, it is likely to be underestimated.

This is the first cross-sectional BT survey carried out in Italy and in southern Europe, reporting up-to-date results on BTV exposure of the sheep population after an epidemic and providing further knowledge on BT epidemiology, compared with the evidences obtained in northern Europe. As the ecological and climatic conditions in European Mediterranean countries (Spain, Greece, Italy and Balkans) are very different from those of northern Europe, our results contributes to a specific understanding of BTV spread at lower latitudes. In the Mediterranean basin, ecological and climatic factors are reported to directly affect *Culicoides* vector population dynamics, such as prolonged summer, mild winter temperatures and slight seasonal fluctuations, determining prolonged favourable conditions for BTV transmission [1]. In addition, the BTV transmission rates could also be conditioned by local temperatures affecting host-vector interaction, as well as extrinsic incubation time, biting rates and vector mortality rates [37]. Consequently, vector density and other vector variables could influence the spread of BTV and could determine different infection patterns in different climatic regions.

The seroprevalences reported in the present study are lower than expected considering the intensive virus circulation registered as assumed from the sampling conducted in outbreak farms and in municipalities where at least two outbreaks occurred. As the aim of the study was to provide useful evidence to support decisions on vaccination strategies and based on the study design adopted, the estimated seroprevalences could likely represent the highest values within sheep farms following the 2013/14 epidemic. Furthermore, having targeted the survey on two selected areas with the highest risk for BTV1 circulation, the estimation of the likelihood of an animal to have a seropositive result was maximised, compared with that which would have been estimated when including the sheep population of the uninfected areas in the analysis.

The higher seroprevalence observed in clinical outbreaks is likely to be related to an increase in local viral circulation due to higher and prolonged viraemias occurring in clinical cases compared to inapparent infected animals. An increase of BTV viraemia could influence the efficiency of host-vector interaction leading to an increase of the BTV1 transmission rate.

The seroprevalences observed in sheep from areas at higher risk (19% in AO area, 26% in IO area) suggest a poor population immunity level acquired even after a BTV1 epidemic. Therefore, the cost and the effort (in the absence of DIVA vaccine) to identify the small number of seropositive animals can be considered worthless and a vaccination campaign could be planned irrespective of a low proportion of these animals.

Inactivated vaccines are expensive and often not readily available [1]. For these reasons, in the last years, no mass vaccination was performed at country level in Europe despite an extensive virus circulation (BTV4 and BTV8 in France, BTV1 and BTV4 in Italy, BTV4 in Balkans). Voluntary or mandatory vaccination limited to Restriction Zones and around outbreaks were performed [38].

The results obtained from this study indicated that Lazio region is an area of low BTV seroprevalence. The criteria for ranking BT animal vaccination was defined in the following order: animals to be moved to non-restricted areas, annual booster for vaccinated animals remaining in the region, vaccination of sheep on voluntary basis to avoid the clinical form and to reduce production losses, vaccination of cattle to reduce the reservoir/amplification of the virus. Prioritizing the vaccination strategy is, therefore, essential.

Relative to the factors that affect the likelihood of infection at individual animal level, living in woodland or semi-natural areas increased the risk of being BTV positive, compared to living in agricultural areas. This result could be explained by the spatial distribution of the *C*. *obsoletus* group that was found to be associated to the former environment [9], even if not confirmed in the multivariable model.

Although the difference between animal-level and within-herd estimated prevalences was minor and the confidence intervals of these two estimates overlap in the regression analyses, living on an outbreak farms was a major risk factor. Proximity to outbreaks the number of outbreaks were also identified as risk factors. These findings are consistent with higher exposure rates for sheep and vectors to BTV infection due to a higher proportion of viraemic animals.

For animal density, cattle are considered BTV amplifiers because of their long-term viraemia [1] and the major role they have in BTV diffusion is confirmed in the present study and in the findings obtained from other studies [39, 40, 41]. The small ruminants’ density was identified as a protective factor. In the present study, it was negatively associated with BTV infection, while in the cited studies it resulted as a risk factor [39, 40, 41]. Nevertheless, no association could be proven in the study conducted by Arenas-Montes et al. [42], while Pioz et al. [43] confirmed a negative association. Therefore, sheep density seems to have a controversial effect on BTV positivity at animal level.

Relative to the sheep herd size affecting the likelihood of being BTV seropositive, there is limited amount of information in the literature. The herd size was positively associated with BTV1 epidemic in Spain [40], while in comparable studies only sheep density in the proximity of the farm was considered. In the present study, BTV infection decreased significantly with the increase of the number of sheep inside a farm. The virus pressure in the vectors and ruminants would decrease due to a dilution effect caused by the increase of the density of susceptible sheep on the farm. Nevertheless, this interesting finding requires further investigation.

## Conclusions

The BTV1 epidemic described in this paper caused limited economic losses, while the estimated cost to perform mass vaccination with an inactivated vaccine was very high. Moreover, other BTV serotypes started to circulate in neighbouring countries. Therefore, the competent authorities decided to exclude mass vaccination. This option is nowadays supported by EFSA opinion [15], which reports that even mass vaccination lasting 3 years and up to 95% of animal population can be inadequate for BT eradication. Nevertheless, considering the low level of seroprevalence found in the population after high BTV1 circulation and the recurrent introduction of new serotypes from the North Africa and the Balkans, the control of endemic and multiserotype BTV infections in southern Europe will continue to represent a challenge in the near future.

## Supporting information

S1 File Raster files used to run ArcGis (ESRI, Redlands, CA, USA) kernel ruminants’ density (Figs 1 and 2). (RAR)
